# The Real Impact of De Novo Bladder Dysfunction After Transvaginal Tension-Free Vaginal Tape-Obturator (TVT-O): A Prospective Study

**DOI:** 10.3390/jcm15145527

**Published:** 2026-07-15

**Authors:** Francesco Plotti, Andi Stermasi, Stefania Rampello, Gianna Barbara Cundari, Federico Liparulo, Arianna Martinelli, Corrado Terranova, Carlo De Cicco Nardone, Daniela Luvero, Federica Guzzo, Nicola Alboré, Roberto Montera, Roberto Angioli

**Affiliations:** 1 Department of Gynecology, Fondazione Policlinico Universitario Campus Bio-Medico, Via Alvaro del Portillo 200, 00128 Roma, Italy; f.plotti@policlinicocampus.it (F.P.);; 2 Research Unit of Gynecology Oncology, Department of Medicine and Surgery, Università Campus Bio-Medico di Roma, Via Alvaro del Portillo 21, 00128 Roma, Italy; 3 Natural Center for Radioprotection and Computational Physics, Istituto Superiore di Sanità, Viale Regina Margherita 29, 00161 Roma, Italy; 4 Department of Physics, University of Rome “Tor Vergata”, Via Della Ricerca Scientifica 1, 00133 Roma, Italy

**Keywords:** stress urinary incontinence, midurethral sling, transobturator tape, voiding dysfunction, OAB

## Abstract

**Introduction**: Transobturator midurethral slings (MUSs) are commonly used to treat female stress urinary incontinence (SUI). Despite their high success rates, postoperative complications such as voiding dysfunction (VD) and de novo overactive bladder (OAB) symptoms may affect quality of life (QoL). This study aims to evaluate patient-reported outcomes regarding bladder dysfunction (VD and OAB) and SUI resolution. **Methods**: Patients undergoing transvaginal tension-free vaginal tape-obturator (TVT-O) for SUI without detrusor overactivity were enrolled from April 2018 to April 2023. Final 12-month follow-up was completed in April 2024. Follow-up at 6 and 12 months included physical examination, stress tests, and a 3-day voiding diary. The Urinary Symptom Profile (USP) questionnaire was administered at 12 months. A VAS score evaluated the subjective bother of voiding dysfunction. Objective cure was defined as a negative stress test at follow-up. Changes in daily leakage episodes were assessed using the 3-day voiding diary. **Results**: Ninety-two patients were enrolled, of whom eight-nine completed the 6- and 12-month follow-up and were included in the final analysis. The objective cure rate was, respectively, 93% at 6 months and 89% at 12 months. The USP questionnaire administered at 12 months showed that the SUI domain score was 0 in 80.9% of patients. De novo urgency was reported in 44% of patients. Urge incontinence and nocturia occurred, respectively, in 33% and 8% of patients. Patient-reported voiding symptoms, defined as LS ≥1, were observed in 58.4% of patients. Among patients with LS ≥1, 38/52 (73.1%) reported tolerable bother (VAS < 5), whereas 14/52 (26.9%) reported clinically important or intolerable bother (VAS ≥5). **Conclusions**: TVT-O was associated with a high objective cure rate; however, patient-reported OAB and voiding symptoms were common at 12 months. These findings support a more comprehensive informed consent process for patients undergoing TVT-O surgery in order to increase patient awareness of the potential development of OAB and VD following the surgical procedure.

## 1. Introduction

Stress urinary incontinence (SUI) is a prevalent and distressing condition characterized by the involuntary leakage of urine during activities that increase intra-abdominal pressure, such as physical exertion, sneezing, or coughing [1]. It represents a common form of urinary incontinence in women and may affect physical, emotional, and social well-being with negative consequences for daily activities, interpersonal relationships, and quality of life (QoL) [2,3]. The reported prevalence of urinary incontinence varies according to age, parity, and assessment methods, with most studies describing rates between 25% and 45% [4]. Among surgical treatments, midurethral slings (MUSs) are widely used for female SUI and have become a standard surgical option because of their minimally invasive nature and high success rates [5,6]. MUS procedures may be performed through retropubic or transobturator approaches. The transobturator route includes the outside-in transobturator tape (TOT) technique and the inside-out tension-free vaginal tape-obturator (TVT-O) technique described by de Leval [7,8]. These approaches have shown comparable efficacy, with a favorable safety profile compared with the retropubic route [9,10]. Nevertheless, despite the overall positive outcomes associated with TVT-O and other transobturator procedures, postoperative bladder symptoms may still occur. Among the most relevant are voiding dysfunction (VD) and de novo overactive bladder (OAB) symptoms, which may influence subjective success and patient-perceived quality of life [11]. De novo OAB is characterized by urgency, frequency, and nocturia, which can occur postoperatively. VD may be characterized by difficulty initiating urination, a sensation of incomplete bladder emptying, or urinary retention, and in some cases may require prolonged catheterization, tape mobilization, or even surgical revision [12]. Prospective studies specifically assessing patient-reported bladder dysfunction after TVT-O remain limited, and previous studies have often focused primarily on objective cure, urinary retention, catheterization, or surgical revision. As a result, milder patient-reported voiding and OAB symptoms may be underrecognized. The novelty of the present study lies in its prospective assessment of postoperative bladder symptoms using symptom-based patient-reported tools, with specific attention to their perceived impact.

The primary aim of this prospective study was to evaluate patient-reported outcomes related to bladder dysfunction, including VD and OAB, following TVT-O surgery. The secondary objective was to assess objective cure of SUI and changes in daily leakage episodes during follow-up.

## 2. Materials and Methods

This prospective observational study was conducted at the Department of Gynecology of our institution. Participants were recruited between April 2018 and April 2023. Each participant underwent baseline, 6-month, and 12-month assessments after surgery. The final 12-month follow-up assessment was completed in April 2024. The study protocol was reviewed and approved by the Institutional Review Board (IRB), and all participants provided written informed consent prior to enrollment, in accordance with the Declaration of Helsinki.

### 2.1. Patient Selection

Female patients presenting with symptoms of stress urinary incontinence (SUI) and deemed eligible for transobturator midurethral sling surgery (TVT-O) were screened for inclusion. Eligibility criteria required a confirmed clinical diagnosis of SUI, suitability for vaginal surgical intervention, and the ability to understand and sign the informed consent form. Patients were excluded if they presented with pelvic organ prolapse greater than stage I according to the Baden–Walker classification, overactive bladder (OAB) symptoms, detrusor overactivity on preoperative urodynamic testing, a history of anti-incontinence procedures, prior pelvic radiotherapy, recurrent urinary tract infections (≥3 episodes/year), neurogenic bladder, any diagnosed neurological or psychiatric disorder, or significant language barriers impeding comprehension of informed consent form. Patients with stage 0–1 prolapse were included because absent or minimal prolapse is generally not considered clinically significant, does not usually require concomitant surgical correction, and is unlikely to independently cause relevant bladder outlet obstruction. Conversely, patients with prolapse greater than stage I were excluded to avoid potential confounding related to clinically significant pelvic organ prolapse.

### 2.2. Baseline Evaluation

At enrollment, a detailed demographic and clinical profile was obtained for each participant. This included age, parity, body mass index (BMI), menopausal status, American Society of Anesthesiologists (ASA) physical status classification, and relevant comorbidities such as cardiovascular disease, diabetes mellitus, neurologic disorders, previous hysterectomy, and prior pelvic organ prolapse surgeries. All patients underwent a comprehensive preoperative work-up, including medical history, general physical examination, urinalysis, and urogynecological evaluation. Vaginal defects were assessed using the standardized Baden–Walker system, while urethral mobility was evaluated via the cotton swab test [13]. The cotton swab test was performed with the patient in lithotomy position. A lubricated cotton swab was inserted into the urethra to assess urethral mobility, with hypermobility defined as an angle change > 30 degrees from the horizontal axis. The diagnosis of SUI was confirmed through a combination of clinical and urodynamic findings: a positive cough stress test performed in both supine and standing positions with a bladder volume of 300 mL, and the exclusion of detrusor overactivity on urodynamic studies. As part of the preoperative assessment, all patients underwent a structured evaluation for lower urinary tract symptoms. Baseline urgency, urge urinary incontinence, nocturia, hesitancy, straining to void, sensation of incomplete bladder emptying, and slow stream were specifically investigated through clinical interview and urodynamic assessment, including uroflowmetry. Patients reporting preoperative OAB or voiding symptoms, or showing detrusor overactivity during urodynamic testing, were excluded from the study. Therefore, postoperative urgency, urge urinary incontinence, nocturia, and voiding symptoms were considered de novo when reported during follow-up. A 3-day preoperative voiding diary was collected from each patient, documenting the frequency and number of incontinence episodes.

### 2.3. Surgical Procedure

All procedures were performed using the inside-out tension-free vaginal tape-obturator (TVT-O) technique, as originally described by de Leval. A Gynecare TVT-O synthetic macroporous monofilament polypropylene midurethral sling was placed beneath the mid-urethra through the transobturator route using an inside-out needle passage. All surgeries were performed by two experienced urogynecologic surgeons (F.P. and R.M.), each with extensive prior experience in TVT-O procedures [8]. Standard perioperative care included intravenous administration of 2 g of cefazolin 30 min prior to incision. Bladder catheterization was maintained until the morning of postoperative day one, at which time spontaneous voiding trials were performed.

### 2.4. Follow-Up and Outcome Assessment

Postoperative evaluations were conducted at 6 and 12 months and included a pelvic examination, cough stress testing, and the repetition of the 3-day voiding diary. At the 12-month follow-up, participants also completed the Urinary Symptom Profile (USP) questionnaire. This tool, developed by the French Association of Urology, assesses symptom severity across three domains: stress urinary incontinence (SUI range 0–9), overactive bladder symptoms (OAB range 0–21), and obstructive symptoms or “Low Stream” (LS) (range 0–9), for a total USP score range of 0–39. Higher scores indicate more severe symptoms. For the purpose of this study, postoperative voiding dysfunction was operationally defined as a USP Low Stream domain score ≥1. This threshold was intentionally chosen to capture any patient-reported voiding symptom, including mild low-stream or emptying symptoms. Therefore, LS ≥1 was interpreted as symptom presence rather than as evidence of clinically significant bladder outlet obstruction. Perceived bother associated with postoperative voiding symptoms was assessed using a visual analogue scale (VAS) ranging from 0, indicating no bother, to 10, indicating the greatest possible bother. A VAS score < 5 was classified as tolerable bother, whereas a score ≥5 was classified as clinically important or intolerable bother. Objective cure was defined as a negative stress test at follow-up. At the 6- and 12-month follow-up visits, pelvic examination was also performed to assess local vaginal complications potentially related to sling placement, including mesh exposure, extrusion, erosion, or signs of local infection. Postoperative voiding assessment was primarily based on clinical evaluation, patient-reported symptoms, and the 3-day voiding diary. Post-void residual volume, postoperative uroflowmetry/Qmax, and postoperative urodynamic testing were not systematically collected during follow-up. However, clinically relevant postoperative obstructive events were recorded, including urinary retention requiring prolonged catheterization, intermittent self-catheterization, tape mobilization, tape division, revision, or removal. Referral to other centers for mesh-related complications and delayed mesh revision or excision during follow-up were also recorded when present. Recurrent urinary tract infections, groin or thigh pain, chronic pelvic pain, dyspareunia, and partner pain were not systematically collected as predefined study outcomes.

### 2.5. Statistical Analysis

Descriptive statistics were reported as mean and standard deviation for continuous variables with approximately normal distributions. Discrete count variables were analyzed as continuous when distributional assumptions were considered acceptable. Categorical variables were summarized as absolute frequencies and percentages. For continuous outcomes measured across multiple time points, comparisons were performed using Wilcoxon signed-rank tests. Binary outcomes assessed longitudinally were analyzed using Cochran’s Q test to evaluate overall differences across time, followed by McNemar’s test for pairwise comparisons when appropriate. Statistical significance was defined as a two-sided *p* < 0.05. The distributions of the SUI, OAB, LS, and total USP scores were assessed using the Shapiro–Wilk test and demonstrated significant deviation from normality. These outcomes were therefore summarized using medians and interquartile ranges and analyzed using non-parametric methods. Paired longitudinal comparisons were performed using the Wilcoxon signed-rank test, while subgroup comparisons were performed using the Mann–Whitney U test or Fisher’s exact test, as appropriate. Rank-biserial correlation was reported as the effect-size measure for Mann–Whitney U analyses, and Wilson 95% confidence intervals were calculated for proportions. Exact numerators and denominators are reported throughout. Subgroup analyses were exploratory, were not prespecified, and were not adjusted for multiple comparisons; their findings should therefore be interpreted as hypothesis-generating rather than confirmatory. All analyses were performed using Python (v3.10.1) and the SciPy statistical library (v1.15.2) [14].

## 3. Results

**Figure 1:**
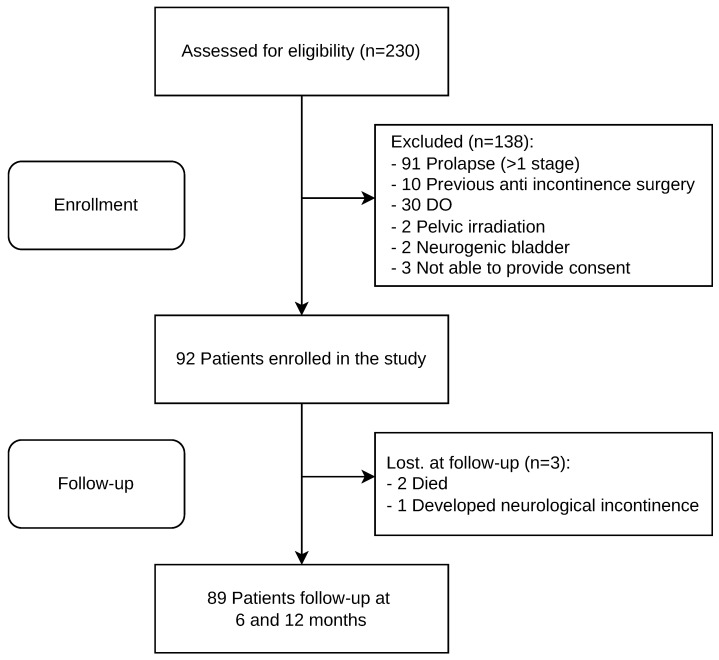
STROBE-style study flow diagram of patient screening, enrollment, follow-up, and final analysis.

A total of 230 patients were initially screened for eligibility between April 2018 and April 2023. Of these, 138 were excluded according to the inclusion and exclusion criteria, and 92 patients were enrolled. Three patients were lost to follow-up; therefore, 89 patients completed both the 6- and 12-month follow-up visits and were included in the final analysis. The study flow diagram is presented in Figure 1.

Baseline characteristics are summarized in Table 1. The mean age was 57.5 years, and the mean body mass index (BMI) was 27.3 kg/m^2^. Menopause was reported in 69% of patients. Comorbidities included diabetes in 9% and cardiovascular disease in 25%. According to the Baden–Walker system, 60% of patients had stage 0 prolapse and 40% had stage 1.

Results of preoperative evaluations including stress and cotton swab tests are detailed in Table 2.

Follow-up cotton swab tests showed a significant decrease in urethral angle from a mean of 69.6±30.2 degrees preoperatively to 31.3±12.4 degrees at 6 months and 33.6±14.3 degrees at 12 months (both *p* < 0.001), with no significant change between the two postoperative timepoints. The stress test was negative in 93% (83/89) of patients at 6 months and 89% (79/89) at 12 months, compared to 0 preoperatively (*p* < 0.001). The 3-day voiding diary revealed a significant reduction in mean daily leakage episodes, from 4.2±1.1 preoperatively to 1.1±0.6 and 1.3±0.7 at 6 and 12 months, respectively (*p* < 0.001).

**Figure 2:**
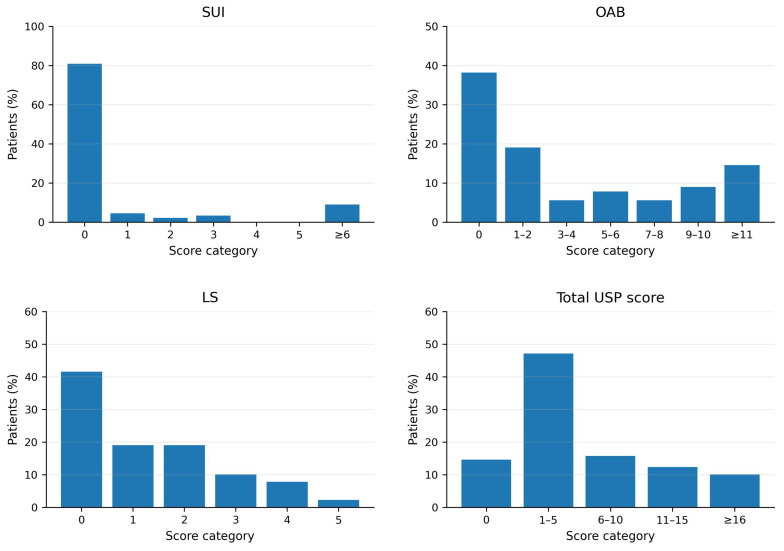
Distribution of Urinary Symptom Profile (USP) scores at the 12-month follow-up. The panels show the proportion of patients within each score category for the stress urinary incontinence domain (SUI), overactive bladder domain (OAB), low-stream domain (LS), and total USP score. The vertical axes represent the percentage of patients within each score category. VAS findings are reported separately in the Results and are not included in this figure.

At 12 months, USP subdomain scores showed marked right-skewed distributions (Shapiro–Wilk *p* < 0.001 for all subdomains and the total score; Figure 2), and are therefore reported as median (IQR) rather than mean ± SD. The SUI domain score had a median of 0 (IQR 0–0), with 80.9% of patients scoring 0. The OAB domain score had a median of 2 (IQR 0–8), with 54/89 patients (60.7%) scoring 0–3 and a smaller subset showing moderate to severe scores. De novo urgency was reported by 39/89 patients (44%), urge incontinence by 29/89 patients (33%), and nocturia by 7/89 patients (8%). The LS (voiding dysfunction) domain score had a median of 1 (IQR 0–2). Voiding symptoms, defined as LS ≥1, were observed in 52/89 patients (58.4%, 95% CI 48.0–68.1%). The total USP score had a median of 4 (IQR 1–10). In an exploratory subgroup analysis, the prevalence of postoperative voiding symptoms was similar among patients with and without previous pelvic surgery: 14/24 patients (58.4%) versus 38/65 patients (58.5%), respectively. No cases of voiding dysfunction were observed in younger patients (≤45 years). Patients with diabetes (n=8) had significantly higher OAB scores than those without diabetes (median 9, IQR 1.75–12.25 vs. median 1, IQR 0–7; Mann–Whitney U =462.5, p= 0.041, rank-biserial r = 0.43). Given the small size of the diabetes subgroup, this finding should be interpreted as exploratory and hypothesis-generating.

Among the 52 patients who reported postoperative voiding symptoms, defined as an LS score ≥1 at 12 months, perceived bother was assessed using the VAS. Overall, 38/52 patients (73.1%) rated their symptoms as tolerable, with a VAS score < 5, whereas 14/52 patients (26.9%) reported clinically important or intolerable bother, with a VAS score ≥5. These findings suggest that the LS ≥1 threshold identified both mild and more troublesome symptoms and should not be interpreted as evidence of urodynamically confirmed bladder outlet obstruction.

The distributions of USP subdomains are summarized in Figure 2. No patient experienced urinary retention requiring prolonged catheterization beyond the planned postoperative catheterization, intermittent self-catheterization, tape mobilization, tape division, tape revision, or tape removal during the follow-up period. No cases of vaginal mesh exposure, extrusion, erosion, local infection, referral to another center for mesh-related complications, delayed mesh revision, or mesh excision were recorded during follow-up.

## 4. Discussion

Midurethral slings represent the gold standard for the surgical management of SUI [15]. Surgical interventions, particularly midurethral slings, have revolutionized the management of SUI, providing a minimally invasive and highly effective solution. Among the various techniques, the transobturator approach has gained widespread adoption due to its favorable safety profile, including reduced risks of complications such as bladder perforations compared to the retropubic technique [6]. Despite the overall success of MUS slings, some postoperative issues such as voiding dysfunction (VD) and the development of de novo overactive bladder (OAB) symptoms remain significant challenges for patients and clinicians.

### 4.1. Voiding Dysfunction

According to the International Continence Society (ICS), VD is defined as abnormally slow and/or incomplete micturition, with heterogeneous symptomatology which can significantly impact a patient’s quality of life [1]. The heterogeneity of VD symptoms and variability in their reporting across studies are key factors contributing to the broad range of reported incidence rates. This variability can be attributed to several factors, such as differences in patient populations, surgical techniques, and the criteria used to define VD. In our study, we focused on patient-reported outcomes regarding VD, as these are directly relevant to the patient’s perception of how the symptoms affect their daily lives, providing a more comprehensive understanding of the postoperative experience. While the incidence of VD varies across studies, research suggests that the transobturator sling procedure is associated with a lower risk of urinary retention compared to the retropubic approach. For example, Morey et al. compared the rates of obstructive voiding complications between transobturator and transabdominal approaches for midurethral slings, finding fewer obstructive complications in the transobturator group [16]. Similarly, Ford et al. found that, compared to retropubic procedures, transobturator slings were associated with significantly lower rates of postoperative voiding dysfunction and urinary retention [5]. The incidence of voiding dysfunction after MUS surgery is influenced by various factors, including the preoperative bladder function of the patient, the type of surgical technique used, and the individual’s response to sling placement. Studies reported in the literature describe a wide spectrum of voiding dysfunction after MUS, making it challenging to define the real incidence of these symptoms. Charalambous et al., in a retrospective comparative study of TVT vs. TVT-O, found 0% urinary retention and 0% dysuria at 12 months follow-up in TVT-O group [17]. In 2009, Aniuliene et al. found 3% of patients with urinary retention at 12 months follow-up after TVT-O [18]. Angioli et al. reported 0% urinary retention at 60 months follow-up after TVT-O [11]. These studies mainly focused on urinary retention, while only a few also reported other possible VD symptoms. Ahn et al. in 2015 found that 2.2% of patients experienced retention requiring additional catheterization and tape cutting for prolonged postoperative voiding difficulty, while 10.5% experienced subjective postoperative voiding difficulty until 3 months [19]. Huang et al. achieved similar results, with 11.2% of patients with voiding dysfunction at 12 months and 10.3% in long term follow-up (5 years) [20]. A recent meta-analysis by Lin et al. reported the incidence of storage and voiding symptoms after transobturator MUS ranging from 0.5% to 15.7% [21]. This highlights the fact that while urinary retention may be relatively rare, other forms of voiding dysfunction, such as difficulty initiating urination or incomplete bladder emptying, are more common and may not always be adequately captured by traditional outcome measures such as urinary retention rates. In the present study, more than half of the patients reported at least one symptom suggestive of VD, as assessed by the USP Low Stream domain. This rate is substantially higher than that reported in most previous studies and meta-analyses; however, this discrepancy should be interpreted in light of the different outcome definition adopted. Most published studies have primarily reported clinically evident or more severe forms of VD, such as urinary retention, need for catheterization, elevated post-void residual volume, or surgical revision. Conversely, our study was specifically designed to capture even mild patient-reported voiding symptoms, which are frequently mentioned during routine postoperative follow-up but may not require medical or surgical intervention and are therefore often underreported in conventional outcome assessments. Accordingly, the 58.4% rate observed in our cohort should not be interpreted as the incidence of severe obstructive complications after TVT-O, but rather as the prevalence of any postoperative patient-reported low-stream/voiding symptom, including mild and tolerable symptoms. This interpretation is supported by the VAS analysis, which showed that although VD symptoms were common, the majority of patients considered them tolerable and to not substantially interfere with daily life. These findings highlight the importance of distinguishing between the mere presence of patient-reported symptoms and clinically burdensome VD, and support the use of patient-centered tools to more accurately characterize the postoperative experience after TVT-O.

No cases of VD were observed among patients younger than 45 years in our cohort; however, given the limited size of this subgroup, no definitive conclusions can be drawn regarding the relationship between age and postoperative voiding symptoms. In our exploratory subgroup analysis, previous pelvic surgery was not associated with a higher prevalence of postoperative voiding symptoms. However, given the observational design and the limited subgroup size, this finding should be interpreted with caution.

### 4.2. De Novo Overactive Bladder (OAB)

The development of de novo overactive bladder (OAB) symptoms after midurethral sling surgery is another critical consideration. OAB, characterized by urinary urgency, frequency, and nocturia, is a common complaint in the postoperative population. The incidence of de novo OAB after MUS surgery varies widely in the literature. Zullo et al. observed that OAB symptoms drop over time, going from 8% at one month to 5% at six months to 0% at twelve months after TVT-O surgery [9]. Teo et al. reported de novo OAB symptoms in 11.3% of patients at 1 year follow-up after TVT-O [22]. Long et al., in a retrospective study, observed that 4.4% of patients developed de novo OAB after TVT-O [23]. In summary, there is no consistent data on the percentage of patients developing de novo OAB at one year after surgery. In our study, the incidence of OAB was considerably higher than what has been reported in the literature. Several factors may contribute to the development of OAB after MUS surgery. These include pre-existing bladder dysfunction, irritation caused by the sling material, and the presence of other comorbid conditions such as diabetes and menopausal status [24]. For example, a study by Wang et al. highlighted that diabetes is an independent risk factor for OAB, likely due to diabetic neuropathy, which affects bladder sensitivity and detrusor muscle function [20]. In our cohort, all patients with diabetes reported postoperative OAB symptoms. Although this finding may be explained by diabetes-related bladder vulnerability, including autonomic neuropathy, altered bladder sensation, and changes in detrusor activity, it should be interpreted with caution because of the small number of diabetic patients included. In predisposed patients, TVT-O may unmask or exacerbate a pre-existing subclinical bladder dysfunction rather than directly cause OAB. Therefore, this observation should be considered hypothesis-generating, and larger studies are needed to clarify whether diabetes represents an independent risk factor for de novo OAB after TVT-O. Some authors have suggested that irritation caused by the sling material itself may contribute to detrusor overactivity, while others have proposed that bladder outlet obstruction due to an overly tight sling may lead to compensatory bladder contractions, resulting in OAB symptoms [25].

### 4.3. SUI Resolution and Patient-Reported Symptom Burden

In our study, we found a substantial improvement of SUI in the majority of patients, with an objective cure rate of 89% at 12 months. This finding is consistent with results from other studies that report similar objective cure rates for TVT-O. For example, Huang et al. reported a cure rate of 92.6% at 12 months [26]. A similar percentage was observed by Karateke et al. in a randomized study, which found an objective cure rate of 86.7% at 12 months follow-up after TVT-O [24,27,28]. The low median SUI-domain score at 12 months further supports improvement in continence-related outcomes. However, the USP was designed to evaluate urinary symptom burden and was not a direct measure of overall patient satisfaction or health-related quality of life. Therefore, the present findings should be interpreted as evidence of improvement in continence-related outcomes rather than proof of overall satisfaction with surgery. The presence of postoperative OAB and voiding symptoms in a proportion of participants further demonstrates that successful treatment of SUI may coexist with new urinary symptoms that could influence an individual patient’s perception of surgical outcome.

Focusing on patient-reported urinary symptoms can be considered a strength of the present study, as these symptoms are directly relevant to the postoperative experience. However, several limitations should be acknowledged. First, the USP questionnaire was administered only at the 12-month follow-up and was not available as a baseline questionnaire. Although preoperative OAB and voiding symptoms were excluded through structured clinical and urodynamic evaluation, the lack of baseline USP scoring represents a methodological limitation. Second, objective postoperative voiding measures, such as post-void residual volume, uroflowmetry/Qmax, and urodynamic assessment, were not systematically collected during follow-up. Therefore, patient-reported voiding symptoms should not be interpreted as urodynamically confirmed bladder outlet obstruction. Third, the present study was primarily designed to evaluate postoperative bladder symptoms, including OAB and voiding dysfunction, rather than the full spectrum of mesh-related complications. Although scheduled postoperative pelvic examination included assessment for vaginal mesh exposure, extrusion, erosion, local infection, and the need for tape revision or removal, other outcomes were not systematically collected. In particular, recurrent urinary tract infections, groin or thigh pain, chronic pelvic pain, dyspareunia, and partner pain were not systematically assessed as predefined study outcomes. No referrals to other centers for mesh-related complications and no delayed mesh revision or excision were recorded during follow-up; however, the absence of systematically collected data on the full spectrum of mesh-related outcomes should be considered a limitation.

## 5. Conclusions

TVT-O was associated with a high objective cure rate for stress urinary incontinence in this prospective cohort. Nevertheless, patient-reported OAB and low-stream or voiding symptoms were common at 12 months when assessed using sensitive symptom-based instruments. Because an LS score of at least 1 may include mild symptoms, these findings should not be interpreted as demonstrating clinically significant obstruction in all affected patients, particularly in the absence of systematic postoperative uroflowmetry, residual-volume measurement, or urodynamic assessment. Preoperative counselling should address the possibility of de novo urgency, urge urinary incontinence, nocturia, voiding symptoms, and the broader recognised complications of synthetic sling surgery. Further studies incorporating baseline and postoperative validated questionnaires, objective voiding measurements, pain and sexual-function outcomes, and longer follow-up are required to clarify the clinical significance and persistence of these symptoms.

## Figures and Tables

**Table 1:** Patients characteristics.

Characteristics	Value
Mean age ± SD (range)	57.5±10
Menopausal status, *n*. (%)	62 (69)
Mean parity ± SD	2±0.7
Mean BMI ± SD, kg/m^2^	27.3±4.1
Mean ASA score	2.2±0.3
No. of cardiopathy, *n*. (%)	23 (25)
No. of diabetes mellitus, *n*. (%)	8 (9)
No. of previous pelvic surgery, *n*. (%)	24 (27)
No. of previous hysterectomy, *n*. (%)	17 (19)
No. of previous prolapse repair, *n*. (%)	7 (8)
Baden–Walker stage:	
Stage 0, *n*. (%)	54 (60)
Stage 1, *n*. (%)	35 (40)

Baseline demographic and clinical characteristics of the study population. Continuous variables are reported as mean ± standard deviation, discrete count variables as absolute values, and categorical variables as number (percentage).

**Table 2:** Comparison between preoperative (baseline) and postoperative (6 and 12 months) data (*n* = 89).

Characteristics	Baseline	6 Months	12 Months	*p*-Value
Cotton swab test	69.6±30.2	31.3±12.4	33.6±14.3	p<0.001 Baseline vs. 6 months
(mean ± SD)				p<0.001 Baseline vs. 12 months
				Not significant 6 vs. 12 months
Stress test				
negative, *n*. (%)	0 (0)	83 (93)	79 (89)	p<0.001 Baseline vs. 6 months
positive, *n*. (%)	89 (100)	6 (7)	10 (11)	p<0.001 Baseline vs. 12 months
				Not significant 6 vs. 12 months
Mean daily leakage	4.2±1.1	1.1±0.6	1.3±0.7	p<0.001 Baseline vs. 6 months
episodes				p<0.001 Baseline vs. 12 months
				Not significant 6 vs. 12 months

Continuous variables are reported as mean ± standard deviation, discrete count variables are reported as absolute values, and categorical variables as number (percentage). Longitudinal comparisons of continuous outcomes were performed using Wilcoxon signed-rank tests. Longitudinal binary outcomes were analyzed using Cochran’s Q test, followed by McNemar’s test for pairwise comparisons.

## Data Availability

The raw data supporting the conclusions of this article will be made available by the authors on request (data are not publicly available due to privacy).

## Abbreviations

The following abbreviations are used in this manuscript:

MUS	Midurethral Slings
SUI	Stress Urinary Incontinence
VD	Voiding Dysfunction
OAB	Overactive Bladder
TVT-O	Tension-free Vaginal Tape-obturator
USP	Urinary Symptom Profile
VAS	Visual Analogue Scale
ASA	American Society of Anesthesiologists
LS	Low Stream
